# Effects of a Personalized Fitness Recommender System Using Gamification and Continuous Player Modeling: System Design and Long-Term Validation Study

**DOI:** 10.2196/19968

**Published:** 2020-11-17

**Authors:** Zhao Zhao, Ali Arya, Rita Orji, Gerry Chan

**Affiliations:** 1 Institute of Communication, Culture, Information and Technology University of Toronto Mississauga Mississauga, ON Canada; 2 School of Information Technology Carleton University Ottawa, ON Canada; 3 Faculty of Computer Science Dalhousie University Halifax, NS Canada

**Keywords:** persuasive communication, video games, mobile apps, wearable electronic devices, motivation, mobile phone

## Abstract

**Background:**

Gamification and persuasive games are effective tools to motivate behavior change, particularly to promote daily physical activities. On the one hand, studies have suggested that a *one-size-fits-all* approach does not work well for persuasive game design. On the other hand, player modeling and recommender systems are increasingly used for personalizing content. However, there are few existing studies on how to build comprehensive player models for personalizing gamified systems, recommending daily physical activities, or the long-term effectiveness of such gamified exercise-promoting systems.

**Objective:**

This paper aims to introduce a gamified, 24/7 fitness assistant system that provides personalized recommendations and generates gamified content targeted at individual users to bridge the aforementioned gaps. This research aims to investigate how to design gamified physical activity interventions to achieve long-term engagement.

**Methods:**

We proposed a comprehensive model for gamified fitness recommender systems that uses detailed and dynamic player modeling and wearable-based tracking to provide personalized game features and activity recommendations. Data were collected from 40 participants (23 men and 17 women) who participated in a long-term investigation on the effectiveness of our recommender system that gradually establishes and updates an individual player model (for each unique user) over a period of 60 days.

**Results:**

Our results showed the feasibility and effectiveness of the proposed system, particularly for generating personalized exercise recommendations using player modeling. There was a statistically significant difference among the 3 groups (full, personalized, and gamified) for overall motivation (*F*_3,36_=22.49; *P*<.001), satisfaction (*F*_3,36_=22.12; *P*<.001), and preference (*F*_3,36_=15.0; *P*<.001), suggesting that both gamification and personalization have positive effects on the levels of motivation, satisfaction, and preference. Furthermore, qualitative results revealed that a customized storyline was the most requested feature, followed by a multiplayer mode, more quality recommendations, a feature for setting and tracking fitness goals, and more location-based features.

**Conclusions:**

On the basis of these results and drawing from the gamer modeling literature, we conclude that personalizing recommendations using player modeling and gamification can improve participants’ engagement and motivation toward fitness activities over time.

## Introduction

A sedentary lifestyle is defined as a lifestyle in which an individual does not receive regular amounts of physical activity, which is becoming a significant public health issue [1]. Various solutions have been considered to encourage a more active lifestyle. Among them, combining exercise with gameplay [2] and the use of wearable trackers to motivate and recommend physical activities [3] have received widespread popularity, but both have user retention issues [4]. This paper addresses the issue of improving long-term engagement with such physical activity recommenders and exercise games.

The popularity of computer games and their engaging nature has created a strong trend to use games for nonentertainment purposes [5]. This trend includes overlapping topics and terms such as gamification (the use of game features and mechanics in nongame applications [6]), serious games (aimed primarily at being an educational yet entertaining tool [7]), persuasive games (games for promoting behavior change [8]), and exergames (a combination of physical exercise with games [9]). In particular, gamification has received significant attention because it can be seen as an umbrella topic covering a range of options from implementing few game elements (such as leaderboards) in regular activities to performing serious tasks as a full game [10,11].

Games and gamified activities are effective persuasive tools for motivating human behavior [12]. Although recent years have seen an increase in persuasive applications designed to promote more active lifestyles [13], studies have suggested that a *one-size-fits-all* approach is ineffective for such persuasive applications because different users are motivated by different persuasive strategies [14] as well as personal reasons such as curiosity and social rewards [15]. There is an increasing demand for personalization as a means of tailoring an experience to individual needs and interests [15,16]. This is particularly the case for persuasive and recommender systems such as those in marketing, education, and health, where retention is as important as initial action [17]. Among different personalization solutions, player modeling in games that aims to understand players to enhance game experience has been an active research topic [18]. It aims to describe a game player’s traits and preferences as well as the players’ cognitive, affective, and behavioral patterns [19] within well-defined structures that allow designers to tailor game contents or goals automatically to suit the needs or preferences of individual players.

Gamification has rapidly emerged over the past years, especially in the area of exercise and fitness [20], as a tool to promote healthy behaviors and maintain an active lifestyle [9]. Researchers have used various gameplays and game features to make exercise and physical activities more engaging and attractive [2,3]. The use of gamification to promote a more active lifestyle can be through either formal exercises performed as games (ie, exergames) or combining games with other physical activities that are not as rigorous as formal exercise (ie, a walk to work). In this paper, we refer to all these cases as gamified physical or fitness activity and use the term “exergame” loosely to indicate the same type of activity. These activities have the potential to help users achieve their fitness goals and increase engagement and pleasure by adding game features to physical activity [12,19].

However, most existing work on gamification and persuasive games in health and wellness are limited because of their use of one-size-fits-all approaches, which have been shown to be suboptimal [21]. As discussed in the next section, there are some research efforts that have used more in-depth personalization, but they are not focused on exergames, and initial attempts at personalization are mostly limited to a small set of static demographic parameters about the user (such as age, gender, and occupation), which makes them more *categorized* rather than *personalized*. In addition, their effectiveness in promoting the desired behavior was mostly evaluated based on a single point of use and feedback (short term). Few attempts have been made to resolve these issues. For example, MyBehavior [22] used a tracking-based and dynamically modified user model to recommend activities, but the system is not gamified and is based on a limited model of daily activity information. A proper combination of a detailed model with features such as personality types, modeling-based personalization, and recommendation with adaptive gamified elements is still missing in the area of exergames, as discussed in more detail in the next section.

To address these research gaps, we propose a comprehensive model for gamified fitness recommender systems that use detailed and dynamic player modeling and wearable-based tracking to provide personalized game features and activity recommendations. We also present the results of a long-term investigation on the effectiveness of our recommender system that gradually establishes and updates an individual player model (for each unique user) over a relatively long period (60 days).

We hypothesized that (1) player modeling based on continuous player activity tracking is an effective approach for personalizing activity recommendations to individual players and (2) combining player modeling and gamification can promote long-term engagement with the system.

On the basis of these hypotheses, we aim to address the following specific research questions in this paper:

1. How can we generate and use continuous player modeling to personalize activity recommendations for each user?
2. Can the combination of player modeling and gamification techniques improve user engagement and experience toward fitness activities over time?

To achieve this, we designed a new player model and a related system architecture for a gamified fitness activity recommender system. To evaluate the effectiveness of the model-driven gamified fitness activity recommender system, we conducted a long-term study on 40 participants and examined the effectiveness of our gamification approach in promoting physical activity in comparison with a control group. We randomly assigned our participants into 4 distinct groups corresponding to 4 experimental conditions:

1. The full group received the application with both gamified features and personalized recommendations (based on player modeling).
2. The personalized group received only personalized recommendations but no gamified features.
3. The gamified group received nonpersonalized recommendations with gamified features added.
4. The control group received generic nonpersonalized recommendations and nongamified features.

The results of a 60-day-long study showed that the idea of generating personalized exercise recommendations using player modeling is feasible. Moreover, it showed that personalizing recommendations using player modeling in combination with gamification can improve participants’ level of engagement and motivation toward fitness activities over time.

Our work includes the following major contributions to the field of fitness recommenders and exergames:

1. We offer a conceptual model and system architecture for personalized gamified activity recommendations using a combination of player modeling, gamifications, and activity tracking.
2. We build a comprehensive player model for personalization that is dynamically updated by continuously tracking player contexts.
3. We design and validate a 24/7 recommender system for personalized activities combined with various gamified elements that adjust to a player’s and environmental contexts.
4. Finally, to demonstrate the feasibility of our approach, we conducted a long-term (60-day) field study with 40 participants to evaluate the proposed system and compare the effectiveness of the 4 experimental groups.

Through the design and development of a new recommender system and a long-term study, we hypothesized and evaluated the effects of gamification and player modeling on physical activity. To the best of our knowledge, this research is the first to link research on player modeling, gamification, and activity recommendation to propose an approach for personalized activity recommendation that is continuously and dynamically updated to reflect users’ changing contexts and states.

### Related Work

#### Gamification

The motivational effects of gamification have been widely studied by researchers. It has been shown that common game elements such as badges, rewards, leaderboards, and avatars are commonly and successfully used to motivate players [10,21-23]. On the other hand, researchers have argued for various areas of improvement in current gamification research and applications, including diversity in themes and context, study duration, and sample size [6,23], increasing motivation by relying on more intrinsic factors [22,24], continuous adaptation [25], and personalization [6]. In the study by Loria [25], a framework for improving the player experience and customizing content generation is proposed by continuously monitoring how players interact during the game by analyzing information such as players’ in-game behavior and players’ social network. Other researchers have also tackled adaptive gamification. For example, Böckle et al [26] identified 4 main elements as the basis for defining meta-requirements and designing principles for building an adaptive gamification system: (1) consider the purpose of adaptivity, (2) define the adaptivity criteria, (3) design the adaptive gamification mechanics and dynamics, and (4) design meaningful adaptive interventions. The researchers applied this framework to the design of a web-based platform for knowledge exchange in postgraduate medical training and reported positive user acceptance, feedback, and increased usage.

Nicholson [24,27] discussed the idea of meaningful gamification (or playification), which focuses on playfulness and activities that make sense to each player and rely on intrinsic motivations as opposed to following specific rules to win. Bertran et al [28] built on this idea and proposed the situational play design, which is a framework for designing context-based and personalized games. Orji et al [14-16], among others, as discussed in a later section, also discussed the idea of player modeling for designing more effective serious and health games. Overall, research in the gamification domain suggests the need for more personalized games that depend on the players’ context and their motivations. The research presented in this paper expands on these ideas and addresses some of the identified needs, such as tracking and understanding the player using a comprehensive individual-level model, personalizing both game features and recommended activities in exergames, and performing long-term studies, within the context of gamified physical and fitness activity recommenders. We discuss the research in these specific areas in later sections.

#### Gamified Physical Activities

Despite their positive effect on promoting an active lifestyle, gamified physical activities face the problem of sustainability (also referred to as player retention here and in other literature) [20,25,26]. Although players may feel excited and motivated to play at first, over time and sometimes quickly, they may lose their willingness to continue. There are studies focusing on the motivation and sustainability of exergames and gamified fitness activities. For example, Campbell et al [21] discussed the concept of everyday fitness games and suggested that for applications that people frequently use in their everyday lives, the design needs to be fun and sustainable as well as adapt to behavioral changes. Macvean et al [29] reported a 7-week study on users’ physical activity, motivation, and behavioral patterns using exergames and suggested that longitudinal studies are necessary for evaluating motivational effects as exergames ensure that the intensity of a user’s behavior is appropriate and sustained. Previous work [30] also showed that based on existing technologies and user needs, the idea of employing wearable activity trackers for gamification of exercise and fitness is feasible, motivating, and engaging. Adding dynamic features could have a positive impact on user motivation toward the gamified exercise system, and the gradual release of application features could increase the user retention rate. It was also found that each user was unique and motivated by different types of game features. Therefore, based on these results, it seems reasonable to generate customized workout sessions to fit different user fitness conditions and interests.

Recently, significant research has been devoted to the design of active games (also commonly referred to as exertion games or exergames) to match the needs of specific groups such as those with disability [28,29] or senior citizens [31] or to use specific technologies such as virtual reality (VR) [32]. Particularly in the context of VR-based exergames for adolescents, research shows that game elements such as the use of rewards, increasing challenge levels, frequent updates, and social or multiplayer options are important aspects for continued engagement in physical activity [33]. Researchers have also investigated design principles for active games [34,35]. Although impressive and invaluable in their findings, these efforts primarily focus on the design of particular games, game features, and actual gameplay mechanisms that are best suited for increasing physical activity for the target group or individual. On the other hand, in 24/7 activity recommenders, the focus is less on designing a particular game and more on gamifying the daily experience and recommending activities based on the daily routine using dynamic player modeling.

#### Player Models

Busch et al [30] indicated that the *one-size-fits-all* approach does not work for persuasive game design. Thus, player-type models could be used when tailoring personalized persuasive systems. One of the most frequently used player-type models is the one developed by Richard [36], who identified 4 player types and proposed that each player has some particular preference for one of the types, which makes them mutually exclusive. Another model is the BrainHex model [37], which is a relatively new model but has been validated using a large pool of participants [38]. In BrainHex, player types were not mutually exclusive. Scores under each category are presented to determine the player’s primary type and subtypes. It also connects player types to the game elements. Moreover, the Hexad model [39], which is of particular interest in our work, is a gamification player-type model created for mapping user personality onto gamified design elements. We considered using the Hexad model in our player model because it specifically targeted gamified systems. It proposes 6 player types, and the player types of individuals are correlated with their preferences for different game design elements. Design guidelines for tailoring persuasive gamified systems to each gamification player type have also been studied [17].

Furthermore, Wiemeyer et al [40] discussed the concept of player experience (with a focus on individual) versus game usability (with a focus on technology) and reviewed various theoretical models that can help understand the player experience. These models are particularly helpful when designing full games as opposed to gamifying everyday activities, which is the goal of this research. However, their insights, such as an integrative multidisciplinary model of player experience, can be helpful in future phases of our research when we focus on the design of game elements. For the work presented here, our focus was primarily on showing how the combination of gamification and player modeling could improve engagement. Better player experiences can be achieved through more complicated models and game features that are beyond the scope of this work.

Personality type also plays an important role in determining people’s fitness tastes [41]. Some people may prefer swimming laps solo, whereas others may enjoy attending a rowdy group-cycling class. These preferences have less to do with people’s physical characteristics and are affected more by personalities. Matching activities to personality type has been shown to have real-world relevance [42]. Research suggests that people who engage in personality-appropriate activities will stick with the activities longer, enjoy their workout more, and have a better overall fitness experience [43]. Brue [44] created a system based on the principles of the Myers-Briggs–Type Indicator (MBTI) assessment. She used MBTIs and reworked them into an easily maneuverable color-coded fitness personality model, the 8 Colors of Fitness, which is also used in our player model. Each color is associated with 2 personality types from the 16 possible MBTI types [45]. For example, blues are loyal, traditional, dependable, and straightforward, whereas greens are nature lovers who seek to quietly merge with the outdoors [42].

Moreover, in recent years, the use of wearable sensors in human activity recognition has become popular [46], in which most of the measured attributes are related to the user’s movement (eg, using accelerometers or GPS), environmental variables (eg, temperature and humidity), or physiological signals (eg, heart rate or electrocardiogram). These data types are naturally indexed over the time dimension, consistent, and convenient to access, which could be used in modeling and predicting a user’s daily activity pattern.

Although there is a significant amount of research on the subject of player modeling, none of the existing studies have examined how to use a comprehensive player model. In addition, no previous research has simultaneously considered both game features and recommended activities in exergames design and investigated whether it is an effective approach over the long term.

#### Personalized Activity Recommendations

Personalized recommender systems for physical activity have been studied by many researchers. For example, Guo et al [47] proposed a system that recognizes different types of exercises and interprets fitness data (eg, motion strength and speed) to an easy-to-understand exercise review score, which aims to provide a workout performance evaluation and recommendation. Although it achieved 90% accuracy for workout analysis, it focuses only on recognizing fitness activities and not personalizing or gamifying them. He et al [48] introduced a system designed to be context aware for physical activity recommendations. It focuses on selecting suitable exercises for individualized recommendations. A smartphone app was developed that could generate individualized physical activity recommendations based on the system’s database of physical activity. The focus of their work is to recommend different types of activities but does not take into account personal details such as proper time, location, and intensity or any gamified elements.

Broekhuizen et al [49] proposed a framework called PRO-fit, which is another example that employs machine learning and recommendation algorithms to track and identify users’ activities by collecting accelerometer data, synchronizes with the user’s calendar, and recommends personalized workout sessions based on the user’s and similar users’ past activities, their preferences, and their physical state and availability. The authors highlighted that many applications nowadays are more focused on tracking user activities but do not provide a recommendation system that would help users choose from activities based on their interests and accomplishment of goals. Therefore, the authors were motivated to design a personalized fitness assistant framework that acts as a motivator and organizer for fitness activities, making it easier for users to create and follow their workout plan and schedule the sessions according to their availability and preference. Compared with PRO-fit, our proposed system provides recommendations in real time throughout daily life as opposed to the prefixed recommendations that are not based on any player or exerciser-type model employed in PRO-fit.

Mittal and Sinha [50] used personal information to recommend general activities such as visiting attractions and shopping. Although not focused on fitness activities or gamification, their notion of modeling user data as the base for recommendation is in line with our proposal. Ni et al [51] used a variety of user data such as daily routine and heart rate to recommend workout routes. Their method is more focused on physical activity recommendations but is limited to recommending routes and does not include gamification elements. Similarly, Rabbi et al [22] proposed MyBehavior, which is a system for tracking users using mobile devices and suggesting food and physical activities. MyBehavior provides personalized and real-time suggestions but is not gamified and does not include an explicit player model. As such, it does not take advantage of full personalization or more engaging features that a game can offer. In line with this, Ghanvatkar et al [52] conducted a comprehensive review of user models used in recommender systems. They highlight that activity profile, demographic information, and contextual data such as location are among the top items to include in user models. In this research, we have defined our player model to include gamer information using Hexad and demographic, activity, and exercise submodels, as suggested by Ghanvatkar et al [52].

### Summary of Research Gaps

As reviewed in previous sections, research on gamified physical activities and related topics has achieved significant results but requires more work to fill the existing gaps. We identified that the main research gap within the context of exergames is the notion of *personalized gamification* (a combination of gamified physical activities with player model–based personalization), including understanding the players and their environment and adapting the game features and physical activities dynamically. None of the existing studies have successfully investigated the effect of gamification and personalization individually with respect to promoting the efficacy of an intervention, specifically a physical activity intervention within a single application. In addition, long-term studies outside controlled environments and real-time activity tracking and recommendation are also frequently missing in existing research on personalization and exergames.

However, the existing body of research provides invaluable insight into recommendations based on real-time tracking, important parameters to include in a player model, and the design of exergames in general. Expanding on existing studies and trying to fill the abovementioned gaps, we propose a conceptual model and system architecture that bring together game elements, dynamic player modeling, and activity tracking to personalize exergames in terms of both game features and recommended activities. We built a comprehensive and dynamic player model for personalization that is continuously updated by tracking the player and offers 24/7 personalized activity recommendations. Finally, we conducted a long-term user study in the wild to evaluate the proposed system.

To the best of our knowledge, although some of the features of our study have been suggested and/or investigated by others, no long-term comprehensive study has been conducted to integrate and evaluate them in real-life exergame apps.

## Methods

### Conceptual Model

Although the existing studies have addressed many aspects of these diverse fields, as discussed earlier, they have not been properly integrated to develop engaging and sustainable exergames. For example, the effect of various game features and continuously adapting the game to player needs and interests have not been investigated in the context of exergames.

In this section, we describe our proposed system architecture and related research methods. This proposal is based on our new conceptual model developed after reviewing related work, consisting of the following principles:

1. Advances in wearable technologies allow game designers to use commercially available activity tracking sensors and mobile devices as a major element of exergames.
2. A game with a static design, no matter how interesting, will lose its attraction after a while. As such, it is important to *add new features* over time to keep players engaged.
3. Although different methods exist for adding dynamic features to games, designers have a limited ability to provide new features constantly, and there is no guarantee that they will be attractive to users. An alternative (or complementary) approach is to dynamically modify the game by *adapting to the player*.

**Figure 1:**
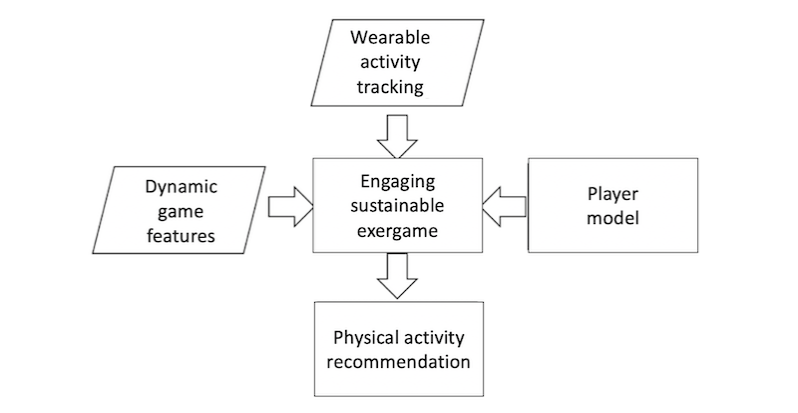
Conceptual model of the proposed system. Components surrounded by parallelograms were explored in our previous work, whereas components surrounded by rectangles are the aim of this research for personalizing physical activity recommendations. Arrows show the connection between the components.

Our conceptual model, which builds on our previous work [28] along with 2 new components, is illustrated in Figure 1.

In our previous work [53], we investigated the effect of using wearable activity tracking in exergames and the long-term effectiveness of using a dynamic game feature–releasing system in sustaining exergames (marked parallelograms). In this paper, we aim to further investigate the gamified features for increasing retention, exploring 2 additional components: (1) player modeling in the personalization of exergames and (2) how to use such a system to generate personalized physical activity recommendations (marked rectangles). The arrows demonstrate how each of the components are related and can directly influence each other. We believe that tracking activity using wearable technology, providing dynamic game features, and detecting a player’s preferences using a player modeling approach can all contribute to creating a more engaging exergame experience, which in turn can generate more personalized physical activity recommendations that players will likely find motivating and satisfying.

### System Design

On the basis of our proposed conceptual model, bringing wearable activity trackers or smartwatches into exergames, dynamically updating game features, and using player modeling for personalization of exergames is being proposed as a solution to the research problem. Therefore, a wearable-based exergame with a comprehensive player model for personalization, recommending customized activities, is proposed as a potential system for further investigation.

The proposed system contains 3 main components: a player model, a recommendation engine, and a game generator. The player model takes different types of user data and predicts user preference for physical activities and finds the proper time and location for recommending activity sessions. It consists of several submodels that cover the user’s general, personality, and daily activity data. The recommendation engine used the output of the player model and generated customized physical activity session recommendations for individual users (including the proper time, location, intensity, and potential type of physical activity). The game generator adds customized game elements to the recommendation and generates the final game content that users can interact with. Wearable activity trackers or smartwatches are used in the system to track the user’s activity and introduce diverse interactions. The combined use of mobile apps and wearable apps will allow users to interact with the system with different modes. The detailed design and development of the system are introduced in the following section.

Overall, a wearable-based exergame system, with a comprehensive player model for physical activity recommendation and game customization, is proposed as a solution to the exergame retention problem.

### Application Design and Implementation

**Figure 2:**
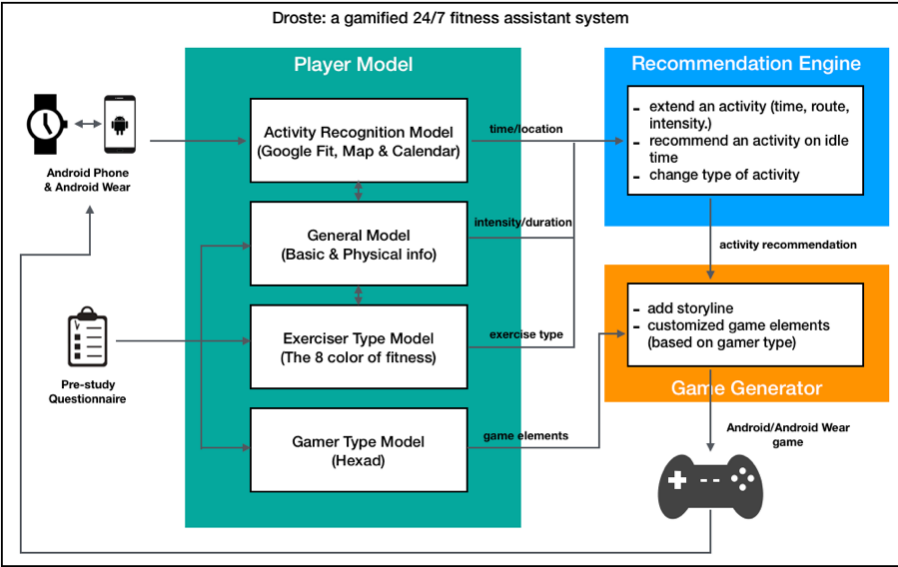
App architecture. How data were collected and transferred to each submodel of the system and how they were used to generate recommendations and game content. Info: information.

On the basis of the proposed system architecture, a Wear OS (formerly Android Wear) application is implemented as the user interface (UI), which tracks the user’s activities and provides gamified fitness recommendations. The overall architecture of the application is illustrated in Figure 2.

In this application, and based on the conceptual model in Figure 1, the player model consists of 4 submodels: (1) an activity recognition model that tracks player activities, (2) a general model that holds basic information about the player, (3) an exerciser-type model that includes information required for recommending activities, and (4) a gamer-type model that is used to choose game features. Each of the submodels is mainly in charge of generating one part of the recommendation, as shown in Table 1. Choosing game features and physical activities are the 2 main personalization options and each has its own submodel. Tracking daily activities is an essential part of the system, which also has a submodel. The fourth submodel holds general player information, such as gender, age, weight, and height.

**Table 1:** The roles of each submodel.

Submodel	Role
Activity recognition model	Time and location
General model	Intensity and duration
Exerciser**-**type model	Exercise type
Gamer**-**type model	Game elements

Although each submodel is designed to generate one particular part of the recommendation, they are still connected to each other to create a more reliable overall recommendation. For example, the exerciser-type model is built for each individual user for recommending different types of activities based on their personalities but it also relies on the general model, which is built based on a user’s fitness and health condition, to exclude those activities that may be suitable for their personality type but not for their health condition. We refer to the theoretical foundations from the Global Recommendations on Physical Activity for Health (GRPAH) [54] to determine proper exercise recommendations in nonpersonalized cases. The GRPAH is an accepted tool approved by the World Health Organization for recommending the appropriate exercise type, duration, and intensity. We use the 8 Colors of Fitness model [44] to suggest different types of activities for the personalized groups. This model is one of the few that uses personality type as the basis of activity recommendations and is suggested by other researchers and practitioners [49,50].

**Figure 3:**
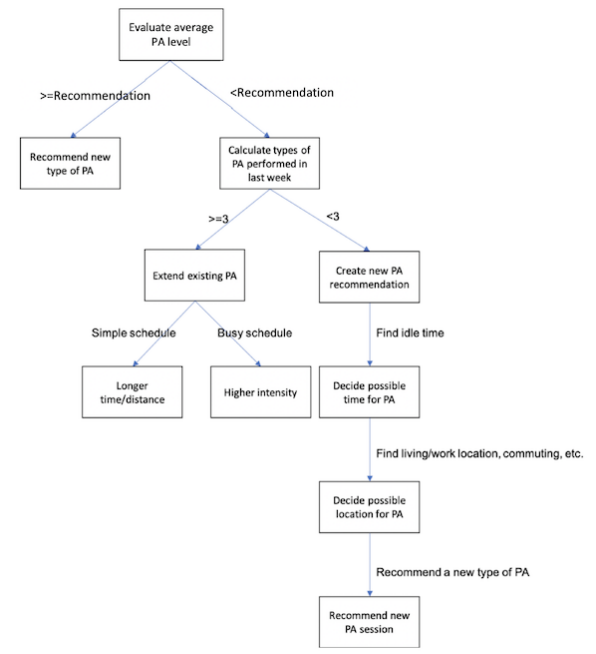
Example of the decision tree used in the recommendation engine (simple version). PA: physical activity.

The recommendation engine is a decision tree–based module that uses all the information generated from the player model to create personalized recommendations for each individual user. It could either extend an existing activity (eg, by recommending a longer exercise time, a longer running path, or appropriate intensity), recommend some activities on the user’s idle time, or simply recommend a different type of activity. An example of a decision tree is illustrated in Figure 3. As the recommendation system for physical activity itself is a relatively complicated topic, we do not consider it as the highest priority for this study. Therefore, we only employed simple decision tree methods to generate basic recommendations (we have ensured that all the recommendations followed the GRPAH guideline for daily physical activities). We are aware that the rationality and quality of the recommended activities would have an impact on user experience. Therefore, after verifying the feasibility of the proposed idea and the roles personalization and gamification performed in this type of system, our research goal will be to investigate the recommender system of physical activities.

The game generator is responsible for adding game elements to the recommendations to gamify the activity suggestions generated by the recommendation engine. The type of game elements to be added is determined by the Hexad player–type model [39]. Our work is also partly based on Orji et al [17], as it adopted a similar Hexad player model. However, as opposed to using the persuasive strategies recommended by Orji et al [17], we used the game elements recommended by the Hexad player–type model, which is more in line with our objective in this work, designing gamified physical activity recommendations. Details of the game and activity recommendations are provided later in this section.

**Figure 4:**
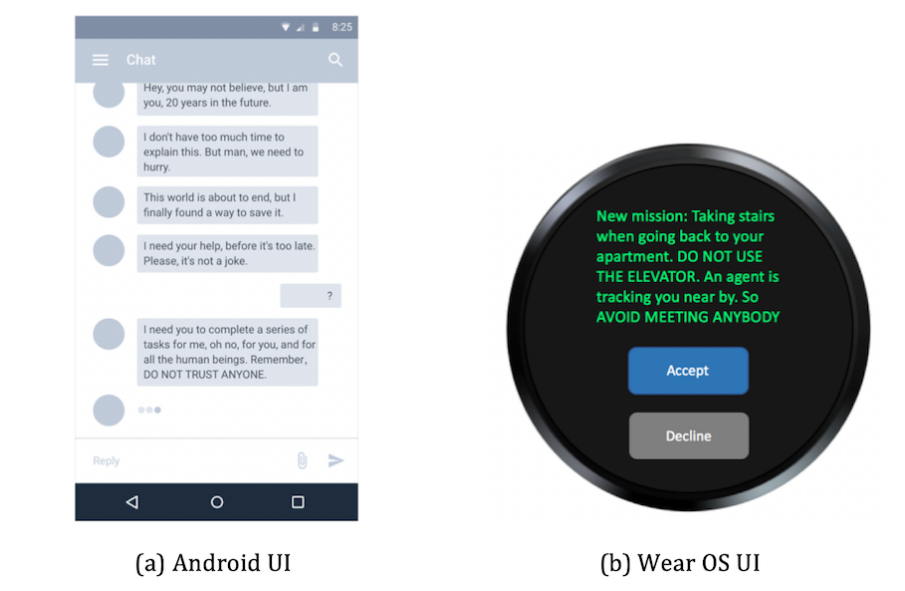
Example app interface and icon. (a) A snippet of one conversation between the system and the user. (b) A display of new mission for the user that he or she can choose to accept or decline. UI: user interface.

A Wear OS app was developed for this study. The app is a conversation-based game in which all the interactions happen in the form of a conversation between the user and the *future self*. The game is based on a story in which a 1-day user receives a message from the future self in 20 years telling him or her that the world is about to end in that future world but only the user can save it by completing a series of tasks. Then, the future self will guide the user through daily activities, which are generated by the recommendation system in a gamified structure. The choice of this game was informed by our need to have a simple design that is capable of incorporating our research requirements but at the same time is not too complicated to develop with many possible confounding factors. We also did not want to introduce various esthetic and design variables to the study that may interfere with our studied research variables and influence our results. For the same reason, we did not try to incorporate our study within an existing game, even though adding these features to games that the user prefers may be another motivating factor in the future. However, it is essential to establish their effectiveness first in isolation. The current system UI was created using a rapid prototyping approach. A pilot study was also conducted before the formal study to ensure that the labels and buttons are clear. The main UI and app icons are shown in Figure 4.

The app tracks the user’s daily activity through Android ActivityRecognition [55] and Google Fit Application Programming Interface (API) [56], which allows up to 6 user activities to be recognized in real time: in vehicle, on foot, running, walking, on bicycle, and still.

The Google Fit API provides encapsulated daily activity–related data such as calories burned, daily steps, and heart rate history (if applicable) tracked by both phone and watch sensors. All the collected activity data, along with their time stamps and location information, are used as input features to train a daily activity model for each individual user by which possible exercise time and location are predicted. As shown in Figure 4, the app is a conversation-based game. We used Wit.ai [57] to generate storylines and to build a bot that can talk to participants and perform some general greetings, tell the time, and talk about the weather. Wit.ai is a tool that uses natural language processing to understand human language and we used its message API to create a chatbot, which aims to understand a user’s intents and lead participants to designed storylines. Moreover, we have included a weather assistant in the system (through the Weather API [58]) to help participants in planning activities around the weather.

**Figure 5:**
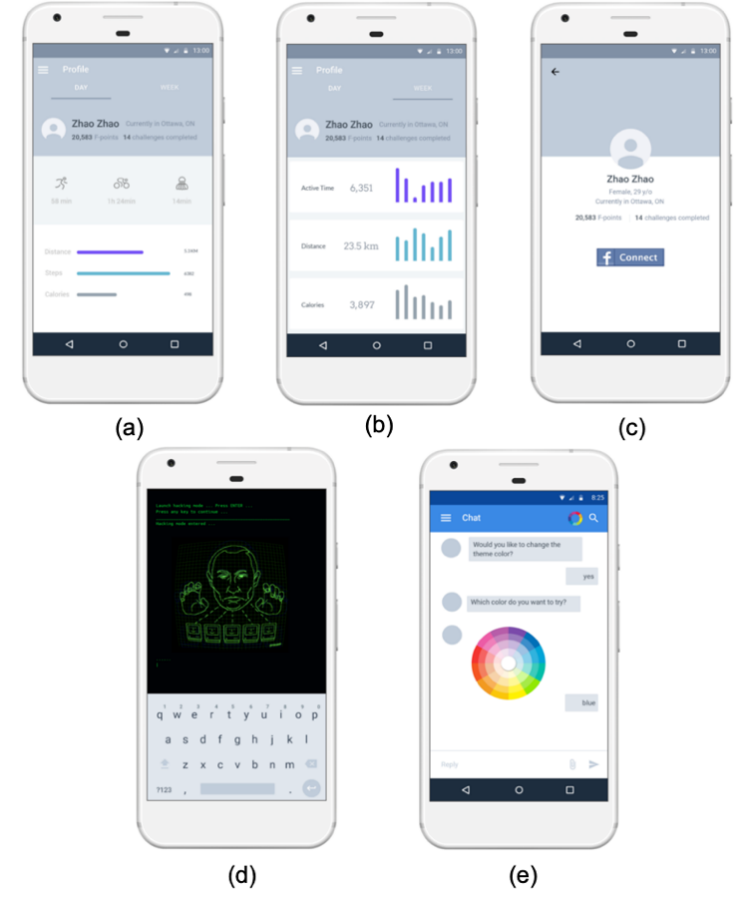
Example game elements: (a) profile in daily view (including points and challenges), (b) profile in weekly view (including points and challenges), (c) connect to Facebook view, (d) hacker mode view, and (e) theme color customization view.

When designing the game features, we employed the Hexad player types [39] and the game design elements guide [17]. Hexad suggests that game design elements are preferred by each player type and we implemented 1 element for each type of user in this study for a personalized game experience (in addition to the game storyline). We integrated the following gamification elements in our game (Table 2). Figure 5 shows some screenshots of example game elements for different player types. If there was a tie in scores between the 6 types, we randomly chose 1 element of the highest score to add.

**Table 2:** The motivation and corresponding game elements added for each type of player.

Hexad type	Motivation	Game element
Socializers	Relatedness	Link to social network
Free spirits	Autonomy and self-expression	Theme color customization
Achievers	Mastery	Challenge
Philanthropists	Purpose and meaning	Game experience sharing
Players	Rewards	Points
Disruptors	Change	Hacking mode

#### Link to Social Networks

*Socializers* are motivated by relatedness. They want to interact with others and create social connections [39]. Therefore, we provided them with an interface for linking the game to their social network as their unique feature so that they could share their game performance or achievements to their Facebook page, team up with those friends who are already in the game, or invite new players to the game.

#### Theme Color Customization

*Free spirits* are motivated by autonomy and self-expression. They want to create and explore the game and prefer features such as unlockable content and customization [17,39]. Thus, we added a feature of theme color customization so that they could customize their game UI by unlocking different themes.

#### Challenge

*Achievers* are motivated by mastery. They are looking to learn new things and want to overcome challenges [39]. Therefore, we added a challenge system for them in our game, in which tasks were assigned to them as challenges.

#### Game Experience Sharing

*Philanthropists* are motivated by purpose and meaning. They want to give to other people and enrich the lives of others in some way with no expectation of reward [39]. For philanthropists, we added a feature for them to share their game experience with other players. A forum-like interface was added to their version of the game in the main screen that allowed them to browse and answer questions of other players. They also receive notifications when there is a new question in the forum.

#### Points

Points have been shown to positively affect *players* [17,39]. They will do what is needed for them to collect rewards from a system. For players, points in our game can be collected and used as virtual currency to buy extra themes or virtual equipment.

#### Hacking Mode

*Disruptors* are motivated by change. In general, they want to disrupt the system [39]. We added a hacking mode for disruptors, in which they can use the command-line interface to access their own game database to make changes to the storyline or delete their records of the game and, eventually, they may *destroy* the system.

As mentioned, the application is a Wear OS game that requires combining the use of both an Android phone and an Android watch to optimize its recognition accuracy and gamified experience. For activity recognition, our app uses watch sensors for better accuracy. However, in many situations, participants may choose not to wear the watch. In those cases, when the watch was not connected, we used the built-in phone sensors instead such that the game could run individually on the phone without the watch. A phone clearly offers more screen space and abilities, such as typing messages, compared with a watch.

### User Study Design

#### Multiphase Research

The proposed conceptual model and the system were evaluated based on a multiphase user study. In our previous work [53], we introduced phases 1 and 2, which can be summarized as follows:

For research phase 1, in-lab user tests of 20 participants were conducted to evaluate the effectiveness of the combined use of games and wearable devices in promoting exercise and to investigate the usability of the proposed approach and the effects of different factors within the system.

In research phase 2, a 70-day user study of 36 participants was designed to verify the hypothesis that adding different game features and gradually releasing them can positively affect user engagement and retention.

In this paper, we present research phase 3, which is a 60-day long-term study with 40 participants, to demonstrate the feasibility and effectiveness of using a player modeling technique in the personalization of exergames.

#### Participants and Groups

A total of 40 participants were recruited locally from the Ottawa area by posters as well as via the web through the Android Wear Forum [59]. Of the 40 participants, 23 were men and 17 were women. Their average age was 26.93 years, with an SD of 6.07 years. We randomly divided our participants into 4 groups based on the versions of the app they received: full (gamified and personalized), gamified only, personalized only, and the control (neither personalization nor gamified, as the control group). Participants were randomly allocated to groups and the distribution with respect to exercise or player type and physical activity level did not seem to be particularly biased (Table 3). Participants’ physical activity levels were collected at baseline before beginning the study.

**Table 3:** Demographical data for the 4 participant groups (N=40).

Characteristic		Participant group			
		Full	Gamified	Personalized	Control
Age (years), mean (SD)		24.93 (7.27)	26.65 (5.58)	27.85 (6.26)	25.78 (5.93)
**Gender, n (%)**					
	Male	6 (60)	6 (60)	5 (50)	6 (60)
	Female	4 (40)	4 (40)	5 (50)	4 (40)
**Hexad user types, n (%)**					
	Philanthropist	1 (13)	1 (11)	1 (10)	1 (11)
	Socializer	1 (13)	2 (22)	1 (10)	2 (22)
	Free spirit	1 (13)	3 (33)	2 (20)	2 (22)
	Achiever	2 (25)	2 (22)	3 (30)	2 (22)
	Disruptor	1 (13)	0 (0)	0 (0)	1 (11)
	Player	2 (25)	1 (11)	3 (30)	1 (11)
**8-color personalities, n (%)**					
	Blue	3 (30)	2 (20)	1 (10)	2 (20)
	Gold	1 (10)	1 (10)	1 (10)	2 (20)
	White	2 (20)	1 (10)	3 (30)	0 (0)
	Purple	0 (0)	1 (10)	1 (10)	0 (0)
	Green	1 (10)	3 (30)	1 (10)	2 (20)
	Red	1 (10)	0 (0)	1 (10)	2 (20)
	Saffron	0 (0)	0 (0)	2 (20)	1 (10)
	Silver	2 (20)	2 (20)	0 (0)	1 (10)
Physical activity level (hours per week), mean (SD)^a^		4.04 (2.35)	3.95 (3.21)	4.82 (2.53)	3.83 (2.92)

^a^Physical activity levels were self-reported at baseline.

To increase the duration in each group and reduce the chance of groups affecting each other, all participants remained in the same group for the entire study duration rather than randomly trying all 4 groups.

The recommendations for the control group and the gamified group were created based on established exercise guidelines and were reasonable recommendations for the general population. To ensure this, we referred to the theoretical foundations from the GRPAH [54] to determine proper exercise recommendations. Our choice for nonpersonalized groups closely follows the one-size-fits-all recommendation method, which has been generally used in most physical activity recommendation applications, such as Apple Watch (recommends a 30-min walk per day) or Fitbit (daily 10,000 steps).

Furthermore, the main purpose of our study was to demonstrate the effectiveness of personalized recommendations. Although we tried to offer a reasonable experience for nonpersonalized groups, the effectiveness of personalization, especially in terms of recommendation, was our hypothesis when other variables are held constant. Therefore, we did not use existing commercial apps for comparison in this study because we tried to avoid bringing in possible extraneous or confounding variables such as esthetic and gameplay features that were not our focus.

**Figure 6:**
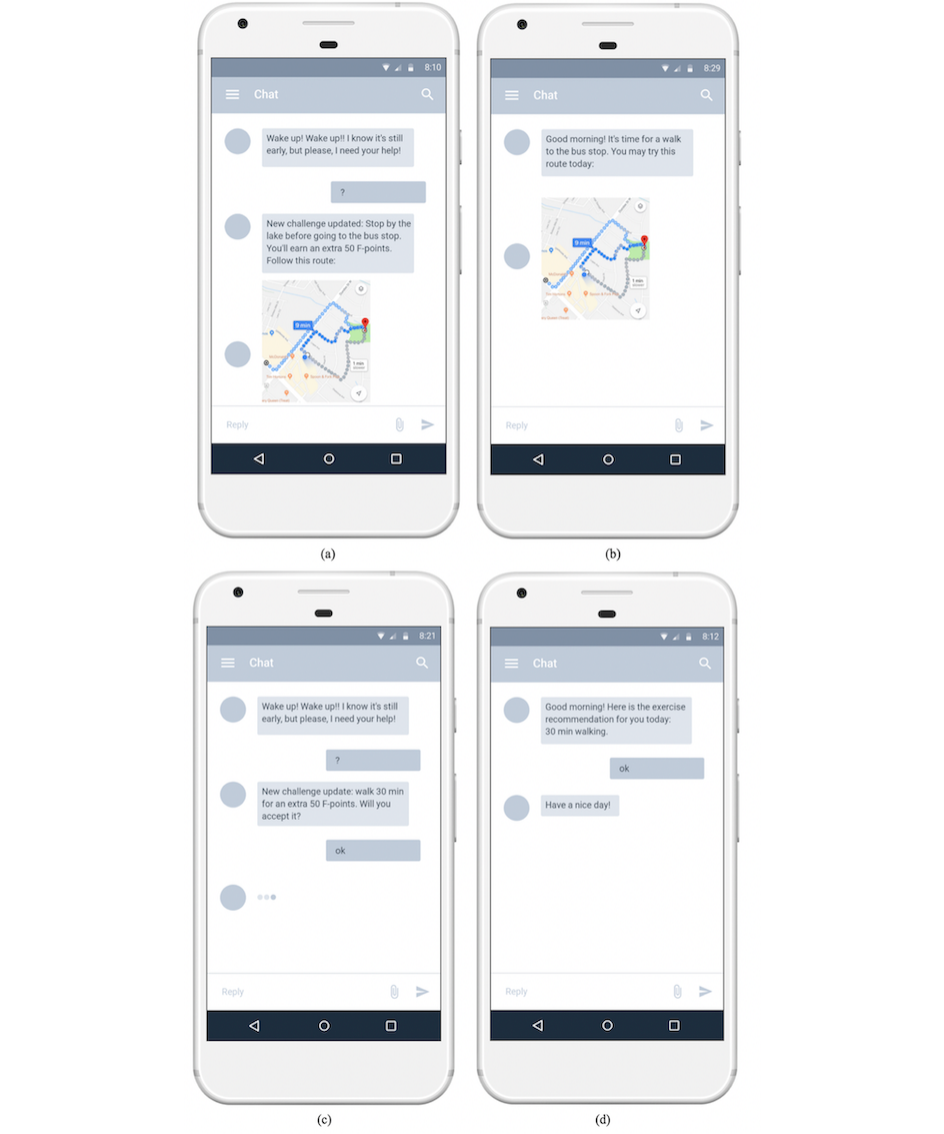
Example of recommendations for different groups: (a) full, (b) personalized, (c) gamified, and (d) control.

Figure 6 shows an example of how recommending the same 30-min walking activity will look for the 4 study groups. The full group received the recommendation through a gamified story (guided by the future self) with the game element of challenge based on their player type of achiever and a personalized walking path. The personalized group also received a personalized route but no game story or elements. The gamified group received no personalized route but had the game story and the game element of points (randomly assigned because no player model was used for the gamified group). The control group received no personalization or gamification as a control group. In the screenshot of the control group, we showed an example of how the weather assistant worked. Note that the example conversations were from screenshots and some details related to the context were not fully displayed.

To control the gamification level between groups, for the gamified group, as there was no player model used, we randomly assigned a game element from Table 2 to each participant to bring them to the same gamification level as the full group. As the members of personalized groups received different game elements based on their individual player model, we decided that a random selection for nonpersonalized groups would be the closest nonpersonalized option.

We also limited the number of personalized recommendations to 2 times a day to eliminate the variability of engagement caused by frequent recommendations. The gamified and control groups (without personalization) received 2 messages per day at 9 AM and 5 PM. We chose these 2 times because 9 AM is the time of day that most of our participants were active. We did not send the notification earlier because we did not want their sleep to be interrupted. We chose 5 PM because most people are off from work at 5 PM. The full and the personalized group received messages based on when they got up and when they left work, as recorded in their individual player model. The results presented in this paper are based on data from a 60-day experiment.

#### Procedures and Data Collection

The study was approved by the research ethics board. We asked participants to complete a prestudy questionnaire before providing them the app (Multimedia Appendix 1). The questionnaire asked demographic questions including age, gender, height, weight, number of hours they spend per week exercising, type of Android Wear owned, and types and duration of playing video games (eg, PC, console, and mobile). Two web-based questionnaires were provided and participants completed them, which provided us with the results to determine their player and exerciser type [60,61]. The app was distributed to participants through the HockeyApp (now Microsoft Visual Studio App Center) [62] after receiving participants’ gamer and exerciser-type results. Application features were selected based on the participant’s player model.

For in-game data collection, we used Google Analytics API [63] to track all participants’ comprehensive in-app behavior data, including screen views and tapped events with associated timestamps. We used Google Fit API to track user daily activity data and a pop-up question asking participants if the recommendation they received that day was useful. The notification was sent to participants every night at 9 PM. For groups with personalized features (the full group and the personalized group), we also asked to access the user’s calendar and location data to be used in recommendations.

A poststudy questionnaire was conducted at the end of the study to evaluate participants’ experiences during the first 60 days (Multimedia Appendix 2). First, we provided 3 general close-ended statements to measure participants’ overall motivation, satisfaction, and preference with the in-game experience. Participants responded on a 7-point Likert scale ranging from 1 (strongly disagree) to 7 (strongly agree). The statements were as follows:

1. I find this kind of application motivating to exercise. 
2. I was overall satisfied with this application.
3. I prefer using this type of application for exercise over regular exercises.

We used the Intrinsic Motivation Inventory (IMI; Multimedia Appendix 3) [64] to assess participants’ level of intrinsic motivation related to the game experience. Furthermore, we used the European Microsoft Innovation Center (EMIC) recommender system evaluation measurement (Multimedia Appendix 4) [65] to evaluate the quality of our recommended activities. We also included open-ended questions to obtain participants’ comments and suggestions to improve the system. By the end of the 60 days, each participant received a Can $10 (US $7.7) gift card as an honorarium to thank them for their participation in the study.

Moreover, we customized the IMI scale to fit the current game context. We did not use *relatedness* and *perceived choice* IMI subscales. *Relatedness* evaluates the experience of doing something with another person, that is, social interactions with a game that can lead to the feelings of relatedness. It is usually used in multiplayer games, which allow for interactions between real players, and was not applicable in our case. *Perceived choice* is often used in situations where a person is given a certain task or activity to complete. In our case, we indicated in the beginning that the users have the full choice of either using or not using our system as well as how to use it. Therefore, this subscale was considered not necessary as the participants were explicitly given full choice. There are different versions of the IMI that have been used in previous studies, which consist of different subscales that are only relevant to their unique context.

#### Data Analysis

For each question in the poststudy questionnaire including general perception, IMI subscales, and EMIC subscales, a one-way between-group analysis of variance (ANOVA) and *post hoc* Tukey-Kramer Honestly Significant Difference (HSD) test [66] was conducted to analyze the main effects among the 4 groups. ANOVA is commonly used to determine whether there are any statistically significant differences between the means of 3 or more independent groups, whereas the Tukey test provides deeper insights into patterns and comparing specific groups [66]. Parametric tests were selected for conducting the analysis because the samples were drawn independently of each other and the shapes of the distributions were normal. The alpha value was set at .05 for all statistical tests.

For other users’ daily log data, such as the number of active users, the number of conversations, the active calories, and the number of useful recommendations, we visualized them along the timeline to see how the pattern differentiated among the 4 groups.

For qualitative data regarding the possible improvement of the system, because our participants’ answers were mostly short and concise, we simply categorized them and reported the most commonly mentioned suggestions.

## Results

### General Information

The participants’ self-reported average hours of exercise per week before the study were 4.16 hours with an SD of 2.96 hours, whereas the average hours per week spent playing video games (including PC, console, and mobile games) were 5.44 hours with an SD of 4.13 hours. The self-reported average active hours increased from 4.16 to 4.58 hours after the study.

Participants could interact with the app through their Android phones or watches. Data show that participants read 53.00% (10,270/19,377) of messages on their phones and 47.00% (9107/19,377) of messages on their watches. They tapped 35.81% (1785/4985) of prompted choices on their phones and 63.99% (3190/4985) on their watches. The results suggest that smartwatches were not only effective and more accurate for tracking activity data but also feasible for some simple interactions such as reading messages and tapping a choice from prompts. Participants tended to interact with watches independently when completing simple tasks and switched to phones when different interactions were necessary (eg, typing messages).

### Case Studies

Below, we present 2 case studies as examples to show how our system recommends activities to different participants in a typical week. If there is any more information or any change in the system found during the week, the recommendations adjust accordingly. Both participants were from the full group receiving activity recommendations in the form of a gamified story.

#### Case Study A

##### Participant Information

Participant A was a female, 26-year-old student, height 5’8’’, weight 61 kg, BMI 20.5 kg/m^2^ (normal weight), no serious health issues, and currently taking no medications. Player type: free spirit; fitness color: white. Our system detected that participant A takes the bus to university every Monday, Tuesday, and Thursday and mostly stays at home for the rest of the week. She goes to a group-cycling class once a week, on Friday evenings, for half an hour. According to the GRPAH, adults aged 18 to 64 years were encouraged to perform 300 min of moderate-intensity aerobic physical activity throughout the week for good health benefits [54]. People with exerciser type of white prefer hiking, running, yoga, cardio, and gym strength training. When accessing her calendar, the system found she had 2 dinner reservations on Thursday and Saturday night, both at 6 PM for the coming week.

##### System-Generated Activity Recommendations

1. Extending the walking distance to bus stops on every school day (both morning and afternoon, overall 45 min of walking per school day).
2. A 30-min walk for non–school days after dinner.
3. A 1-hour home yoga session on Tuesday 7 PM when the user is generally not active.
4. A hiking morning on Saturday in a nearby park.

##### Player Type–Based Game Features

The player type of free spirit was assigned the game feature of *theme color customization*. Thus, the reward of completing recommended activities for participant A was to unlock different theme colors.

#### Case Study B

##### Participant Information

Participant B was a male, 35-year-old, software developer, height 5’11’’, weight 75 kg, BMI 23.0 kg/m^2^ (normal weight), no serious health issues, and currently taking an over-the-counter pain reliever for his back pain. Player type: achiever; fitness color: red. Our system detected that participant B drives to work every Monday, Tuesday, Thursday, and Friday (15-min drive). On Wednesday, he works from home. He plays basketball every Wednesday night from 8 PM to 9 PM and every Saturday morning from 9 AM to 11 AM. Exerciser type of reds prefer exercises such as basketball, tennis, racquetball, in-line skating, frisbee, mountain biking, soccer, and skiing. Our system found that participant B was almost as active as recommended by the GRPAH, but the type of activities he performed was limited to basketball.

##### System-Generated Activity Recommendations

1. A 1-hour tennis or racquetball session on Wednesday night instead of basketball.
2. A daily 15-min walk after work.
3. A 60-min walk (in a nearby park) on Sunday morning.

##### Player Type–Based Game Features

The player type of achiever was assigned the game feature of challenge. Thus, the system provided recommendations to player B in the challenge style.

### Overall Motivation and Satisfaction

Figure 5 shows the averages and SDs of the scores for the first 3 general questions assessing participant motivation, satisfaction, and game preference. The asterisk indicates significant results found between groups.

**Figure 7:**
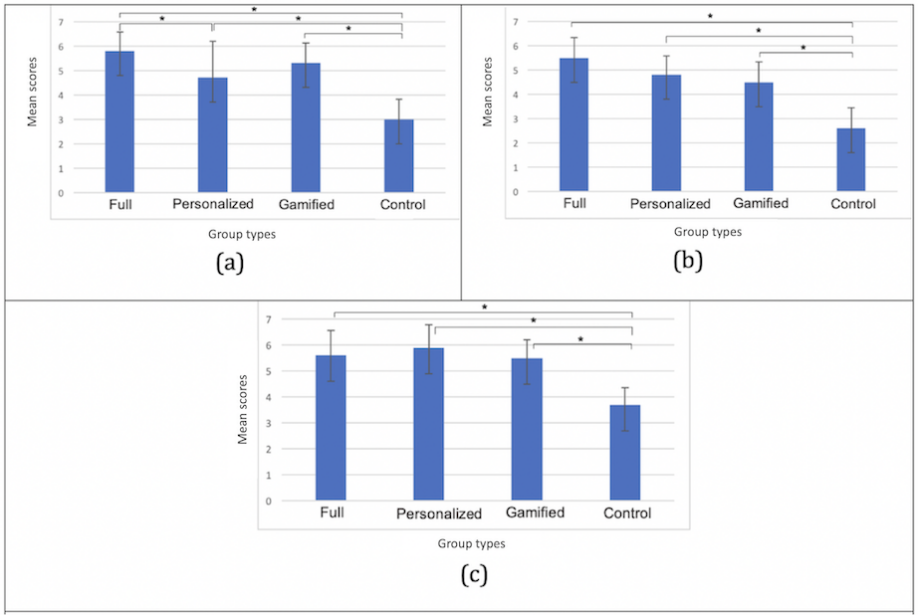
Results for poststudy questions 1, 2, and 3. (a) Overall motivation, (b) overall satisfaction, and (c) overall preference over regular exercise.

The results show that there were statistically significant differences between groups as determined by one-way ANOVA for overall motivation (*F*_3,36_=22.49; *P*<.001), satisfaction (*F*_3,36_=22.12; *P*<.001), and preference (*F*_3,36_=15.0; *P*<.001). *Post hoc* comparisons using the Tukey HSD test indicated that for all 3 questions, the mean score for the full, personalized, and gamified groups was significantly different from that for the control group, respectively. This means that, in general, both gamification and personalization have positive effects on participants’ motivation, satisfaction, and preference, as seen in the groups full, personalized, and gamified compared with the control group. Moreover, for motivation, the mean score for the full group (mean score for full group [MF] 5.8, SD for full group [SDF] 0.79) was significantly different from that of the personalized group (mean score for personalized group [MP] 4.7, SD for personalized group [SDP] 1.5). Statistically significant pairwise comparisons are also marked in Figure 7. This means that gamification can also add more motivation to a personalized fitness recommendation system, as seen between the full group and the personalized group in motivation. It should also be noted that the distribution of the dominant player types across the 4 different groups could have influenced these results, as some player types may have had a stronger preference for gamification or personalization in general. However, the distribution with respect to player types did not seem to be particularly biased (Table 3).

### IMI Subscales

**Figure 8:**
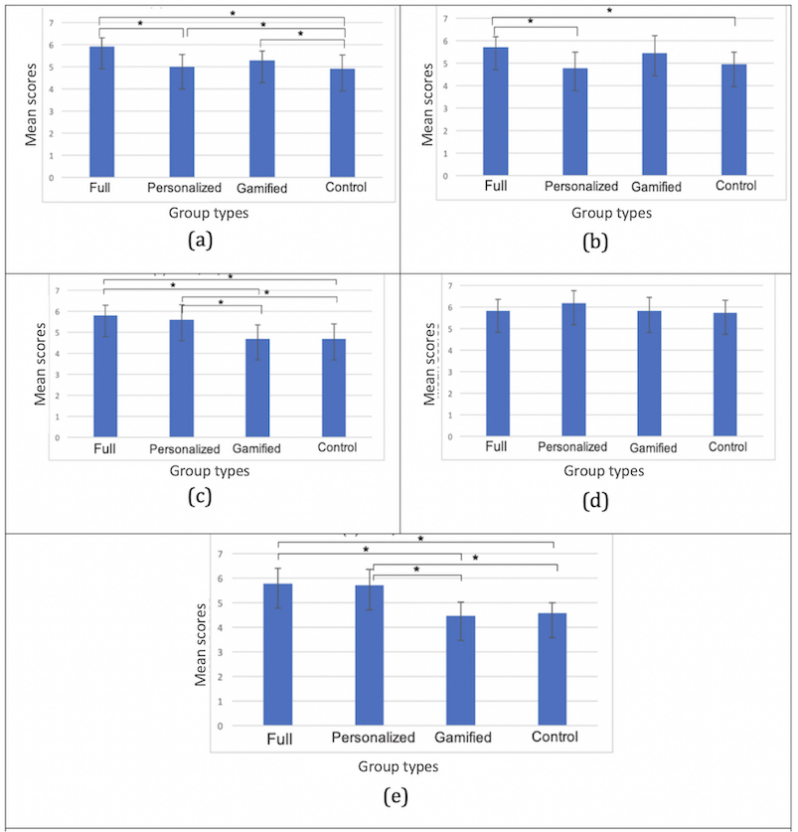
Averages and SDs as evaluated by the Intrinsic Motivation Inventory. From top to bottom: (a) interest or enjoyment, (b) perceived competence, (c) effort or importance, (d) pressure or tension, and (e) value or usefulness.

Figure 8 shows the average and SDs of the scores for each IMI subscale question. The results show that there were statistically significant differences between groups as determined by a one-way ANOVA for interest or enjoyment (*F*_3,36_=24.24; *P*<.001), perceived competence (*F*_3,36_=4.60; *P*=.007), effort or importance (*F*_3,36_=8.01; *P*<.001), and value or usefulness (*F*_3,36_=15.90; *P*<.001).

The Tukey-Kramer HSD test results indicated that for interest or enjoyment, the mean score for the full, personalized, and gamified groups was significantly different from that for the control group. Moreover, the pairwise comparison result showed that MF (MF 5.9, SDF 0.40) was significantly different from the personalized group (MP 5.0, SDP 0.56). For perceived competence, significant differences were found between the full group (MF 5.7, SDF 0.46) and the personalized group (MP 4.8, SDP 0.72) as well as between the full group and the control group (mean score for control group [MC] 5.0, SD for control group [SDC] 0.54). For effort or importance, significant differences were found between the full group (MF 5.8, SDF 0.47) and the gamified group (mean score for gamified group [MG] 4.7, SD for gamified group [SDG] 0.67); between the full group and the control group (MC 4.7, SDC 0.70), the personalized group (MP 5.6, SDP 0.73), and the gamified group; and between the personalized group and the control group. For value or usefulness, significant differences were also found between the full group (MF 5.8, SDF 0.63) and the gamified group (MG 4.8, SDG 0.55); between the full group and the control group (MC 4.6, SDC 0.41), the personalized group (MP 5.7, SDP 0.63), and the gamified group; and between the personalized group and the control group. The pairwise comparison significance is also marked in Figure 8.

The IMI results indicate that gamifying the exercise increases players’ interest in and enjoyment of the personalized recommendation system (significant between the full group and the personalized group in interest or enjoyment). Personalization contributes more toward promoting effort or importance as well as value or usefulness compared with gamification (significant between the personalized group and the gamified group).

### EMIC Recommender System Evaluation

**Figure 9:**
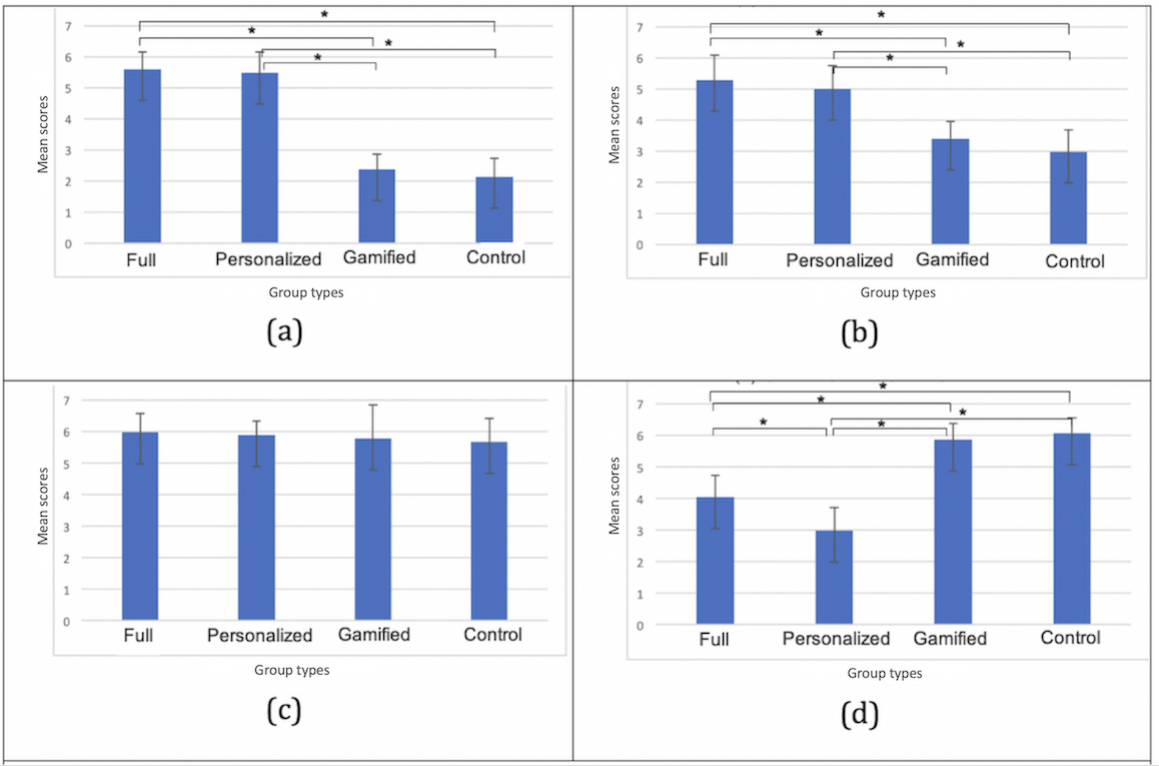
Average and SD of European Microsoft Innovation Center recommendation. From top to bottom: (a) perceived recommendation quality, (b) perceived system effectiveness, (c) general trust in technology, and (d) system-specific privacy concern.

Figure 9 shows the averages and SDs of the scores for each EMIC subscale (under perceived recommendation quality, perceived system effectiveness, general trust in technology, and system-specific privacy concerns). The results showed that there were statistically significant differences between groups as determined by a one-way ANOVA for perceived recommendation quality (*F*_3,36_=108.77; *P*<.001), perceived system effectiveness (*F*_3,36_=26.52; *P*<.001), and system-specific privacy concern (*F*_3,36_=58.37; *P*<.001).

The Tukey-Kramer HSD test results indicated that for both perceived recommendation quality and perceived system effectiveness, the mean scores for the full and personalized groups were significantly different from the gamified and control groups that were not personalized. For system-specific privacy concerns, the mean scores for the full group and the personalized group were also significantly different from the gamified group and the control group because, for nonpersonalized groups, we did not ask to access participants’ personal data (except Google Analytics for in-app tracking). Moreover, a significant difference was also found between the full group (MF 4.1, SDF 0.69) and the personalized group (MP 3.0, SDP 0.73). Statistically significant pairwise comparisons are also marked in Figure 9 using asterisks.

Our results suggest that our system is effective in providing daily fitness recommendations to participants (comparing the full group with the gamified group and the personalized group with the control group) with respect to both perceived recommendation quality and perceived system effectiveness. We also found that, as expected, participants were concerned about privacy when the system had a player model and asked for more permissions to access their personal data (comparing the full or personalized and gamified or control groups). On the other hand, gamification reduced some of the concerns (significant difference found between the full and the personalized groups). Note that for the system-specific privacy question, a higher score indicates less concern. Privacy concerns are important yet beyond the scope of this work. Yet, we believe that the noticed effect of gamification can be of value in future research and design.

### Daily Statistical Data

**Figure 10:**
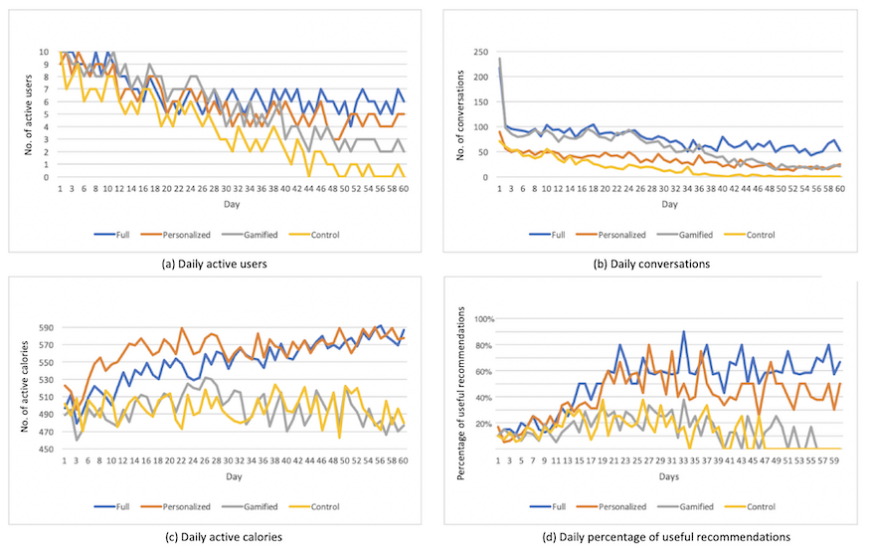
Daily statistical data showing patterns of daily active users, conversations, or active calories and percentage of useful recommendations along the timeline.

As mentioned earlier, we used Google Analytics API to track participants’ comprehensive in-app behavior data and we used Google Fit API to track user daily activity data, including steps and calories burned. Figure 10 shows some daily statistical data: the number of active participants of all 4 groups during the 60 days of study (a), the daily total number of conversations sent to the system (b), the daily average active calories burned excluding basal metabolism (c), and the daily number of self-reported useful recommendations (d).

From Figure 8, we can see that for daily active users (a) and daily conversations (b), there is an overall descent in trends appearing as time grows for all 4 groups. Among them, the full group maintained a relatively higher value compared with the other 3 groups and participants in the full, personalized, and gamified groups interacted with the system more than the control group (Figure 10). With respect to the daily active use and daily conversations (Figure 10), when comparing the personalized group and the gamified group, we can see that the value of the gamified group was higher than that of the personalized group in the early phase of the study but was surpassed by the personalized group in the late phase of the experiment (around 35-40 days). These results indicate that both personalization and gamification could have a positive impact on promoting participants’ engagement with the system. However, although gamification could bring more interactions in the short term (within 1 month), personalization could lead to a more sustained engagement (over a longer time). Note that Figure 10 shows that the control group was not active during the last week of the study. This only indicates that they did not open the app but they still received recommendations as usual (pop-up notifications). Physical activity data were also collected from the Google Fit API without opening the app.

For active calories (Figure 10), we can see slight ascent trends for both the full group and the personalized group and flat trends for the gamified group and the control group. The full group began with a lower average calorie burden compared with the personalized group and then showed an almost equal value near the end. These results indicate that personalization could have a positive impact on promoting actual physical activity, whereas exclusive gamification may not. Adding gamified elements to personalized recommendations in the earlier phase (when the player model was not well established yet and the recommendation quality was not steady enough) may negatively affect the amount of physical activity people performed, which requires further research. Note that the active calorie measures the calories burned during fitness activities. Basal metabolic parameters were excluded.

For the percentage of useful recommendations, Figure 10 (calculated by the daily number of *useful* replies divided by daily active users), the percentage of the full group and the personalized group increased in the first half of the study and then remained flat, with the full group remaining slightly higher than the personalized group. The increase in the full group and the personalized group can be attributed to the continuously updating player model that will improve recommendations over time. The gamified group and the control group (without player model) showed descending trends approaching zero. The results suggest that our system is able to generate useful fitness recommendations by using a player model, and participants considered the recommendation more useful when gamification elements were added.

### Player Types

In this study, we did not find any significant difference in terms of different player or exerciser types. Although we had a limited sample size for conducting a meaningful statistical analysis, there were still some interesting findings worth mentioning, which may help inspire future research in this area. Table 4 shows the distribution of the combinations of player and exerciser types of our participants.

From Table 4, we can see that certain player types and exerciser types were highly related. For example, we have 5 participants in total with the exerciser type of silver, with 4 of them belonging to the player type of free spirit. Similar relations are shown between the player type of socializer and the exerciser type of purples. This indicates that users’ preferences toward game elements and exercise types may be linked. This idea could be used to further improve the personalization of the exercise and game experience but requires further research with a larger sample size.

**Table 4:** Distribution of the combination of player or exerciser type (N=40).

The 8 colors	Achiever	Player	Socializer	Philanthropist	Disruptor	Free spirit
Blue	3	3	0	2	0	0
Gold	1	1	1	2	0	0
White	2	1	0	1	0	2
Purple	0	0	2	0	0	0
Green	0	1	3	2	0	1
Red	2	0	0	1	1	0
Saffron	1	0	0	0	1	1
Silver	0	1	0	0	0	4

**Figure 11:**
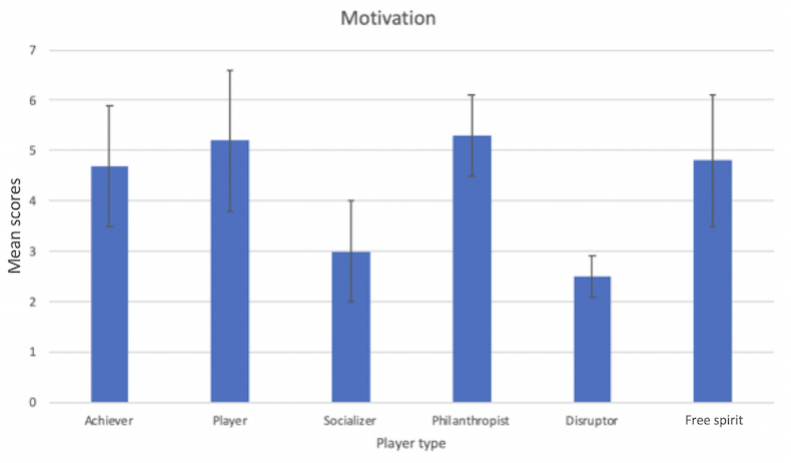
Overall motivation for different player types.

Figure 11 shows the overall motivation for participants belonging to different player types. Although we were not able to run a valid statistical analysis based on the small sample sizes, we saw that the player type of socializer and disruptor experienced lower overall motivation compared with the other 4 player types. This may indicate that the game features and experience we provided to the player type of socializers and disruptors had more room for improvement.

**Figure 12:**
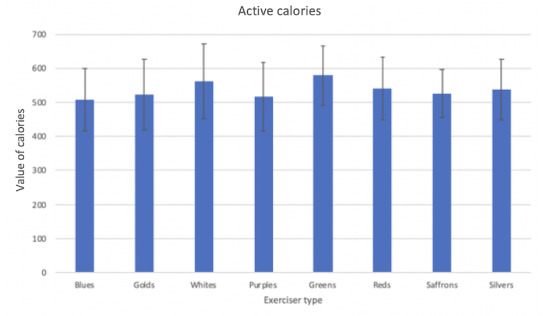
Average active calories for different exerciser types.

Figure 12 shows the average active calories burned for participants belonging to different exerciser types. For the same reason of small sample sizes, we could not run a valid statistical analysis. However, we found that the fitness colors of whites and greens were relatively more active during the study. We checked their motivation as well as their self-reported recommendation quality and found that both were at the same level as the other 6 exerciser types. When looking at the 8 Colors of Fitness activity suggestions (Multimedia Appendix 5), we found that the activity of hiking was the main variable that may lead to the result and it was only recommended for the exerciser type of greens and whites. This indicates that hiking might be an effective activity that makes people consume more calories, which could be further investigated.

### Qualitative Results

**Figure 13:**
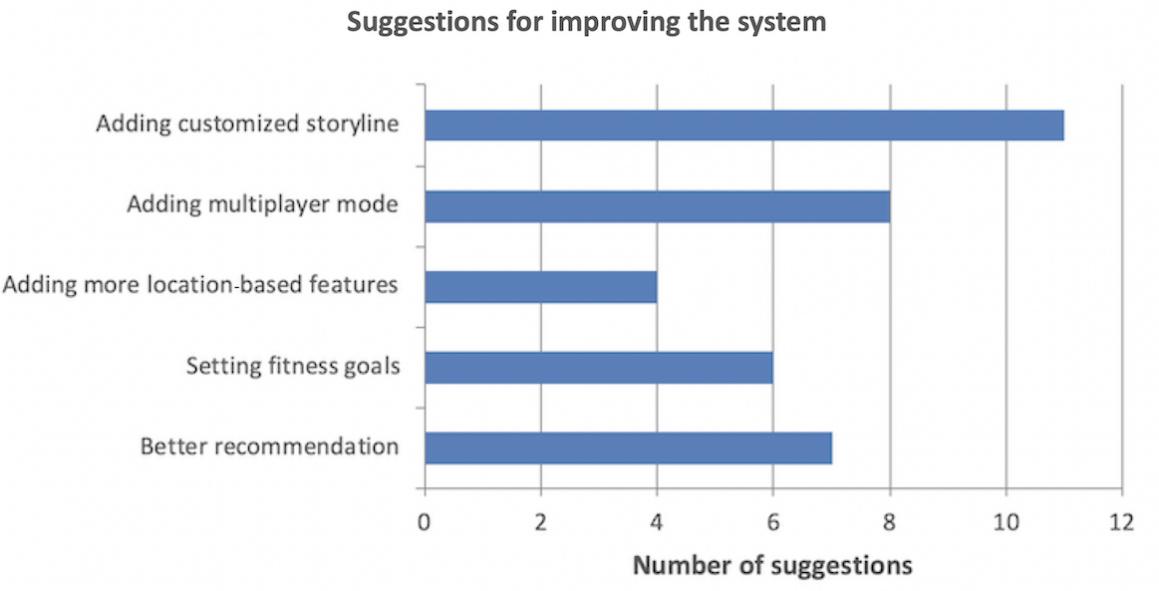
Number of main suggestions received from open-ended questions for improving our system.

One open-ended question was asked of each participant at the end of the poststudy questionnaire to collect their general feedback (Multimedia Appendix 2). We received many comments and suggestions on how to improve our system, which mainly focused on 5 aspects as shown in Figure 13. It shows that a customized storyline was the most requested feature, followed by multiplayer mode, more quality recommendations, a feature for setting and tracking fitness goals, and more location-based features. These feedback laid the foundation for planning our future work in this project.

## Discussion

Overall, in this 60-day user study, we verified our hypotheses that (1) it is feasible to generate personalized exercise recommendations with player modeling and (2) the combination of player modeling and gamification could enhance users’ engagement with the system as well as promote actual physical activity. Specifically, gamification was found to promote engagement, but only in the short term, as seen in the gamified group where the members were engaged early on. However, as the experiment moved on, the trend changed and the personalized group became more engaged. This can be attributed to the player modeling aspect in that it requires time to get to a minimum level of precision in reflecting a player’s characteristics before it can offer reasonable recommendations. Player modeling helped sustain the activity level in the long term. This suggests that activity recommendation based on player modeling can be an effective and promising approach for creating personalized fitness experiences over longer periods, whereas gamification can help attract the users and create the initial interest.

Our research was motivated by the need to keep players engaged and motivated in exergames. We were inspired by previous work that suggested a more player-centric and personalized approach to game design and gamification [6,17,28,67] to increase player engagement and the overall effectiveness of the intervention. We extended these ideas to exergames, combining them with the notion of real-time activity tracking and recommendation as suggested by others [22,47-49] to develop a new theoretical dynamic and individual-level model that brings together various game elements that can help solve the player retention problem. The presented results have direct implications for the design of fitness assistants and potentially other recommender systems.

### Gamification Is Good but Not Enough!

Previous work by authors and other researchers has shown the potential value of gamification to increase engagement, but they have also highlighted the issue of retention. Players tend to leave the game once it is well experienced. Although adding new features can be a reasonable way of keeping participants engaged, it is difficult and costly to implement because of constant designing and upgradation. The ability to understand participants and their dynamic life and provide gameplay features that match the participants’ activities can be a way to introduce change and novelty when maintaining the development cost under control.

### Player Modeling: Personalization Versus Categorization

The idea of categorizing participants to provide them with customized service is appealing but ignores individual differences, which are often significant. The availability of personal data, as a result of various methods of collecting information, suggests that the participants can be understood as individuals and not members of a category. This true personalization allows a new level of customization that will potentially offer participants a much more appealing and effective experience. Our results show the potential relevance of this idea to the field of fitness assistants. The more we understand the user, the more personalized our recommendations will be, which will, in turn, result in more effective recommendations. Developing a comprehensive model that involves various user characteristics (from personality type to daily routines) can help understand the user properly.

Furthermore, the idea of personalization versus categorization is also related to differences in player types and player traits. Although earlier works have attempted to classify players into single types (eg, Bartle [68] and the BrainHex model [36]), more recently, researchers have examined the effectiveness of trait-oriented models for understanding player choices in games [69-71]. Trait-oriented models are preferred in recent studies because an individual is rarely motivated by a single factor and because of their applicability to game user research in that they aim to characterize players using a set of scores rather than categorizing players into a single type. In this study, we decided to use the dominant player type as evaluated by the Hexad model rather than considering all 6 scores because we wanted to control the variable by adding only 1 additional element to each user; therefore, we could make sure it is the gamification itself that affected the engagement, without interfering with the amount of it. A future study can explore the effects of considering the full range of scores.

### Adaptive and Continuous Modeling

Although many games and other applications rely on a certain user model, in most cases, this is done as a one-time static decision assigning the user to a certain group. Our study shows the value of not only having a more comprehensive personal model but also allowing it to evolve and adapt using ongoing data from the user. This constantly tunes the model and makes recommendations more effective. Using such adaptive and dynamic models can enhance the performance of such applications, and we recommend that designers consider it when possible.

### 24/7 Recommendation

Fitness and health are not limited to the gyms. Being active is a lifestyle; therefore, activity recommendations should not be limited to a particular time. In the absence of a dedicated personal trainer, an intelligent fitness assistant equipped with a detailed player model can offer 24/7 recommendations for being active that considers various user contexts. Our results show the potential value of this approach, which can be improved with more comprehensive personal data and a better database of activities and gameplay features. Although our system provided all-day and continuous modeling and recommendation, it is worth noting that the participants did not wear the activity trackers during sleep and we did not track any sleeping activities. As such, although the system was able to perform nonstop, in practice, it was paused during sleep times (night or day).

### Limitations

There were certain limitations in the proposed system and the performed study, some mentioned by the participants, which we believe were not critical enough to significantly affect the findings but are still worth noting and improving in future work.

We relied on a simple game that we designed ourselves with a simple story or gameplay. This may have negatively affected the players’ attraction and engagement. The game could be designed through a more rigorous process or we could somehow allow customization and choice or potentially use another existing game. There was also no multiplayer option, which ignores the social aspects of gaming and active lifestyle and could negatively affect the level of user engagement. When designing different gamified features for different types of players, we assigned only 1 game element to each type of player. This may not be adequate for targeting individual participants.

The 8 Colors of Fitness system (Multimedia Appendix 5) [44] was used as a model to suggest activities. This system was used because the research group did not find any other alternatives and needed to rely on a fairly acceptable method. This system is by no means ideal and has its own limitations. It can be replaced with any other method, such as other models, an interactive trainer, or a trained expert system.

We used the Android Activity Recognition API for activity tracking and prediction in this work. This API is only able to recognize 6 simple physical activities. For more complex daily activities, we required manual labeling from participants within the conversation. This may bring complexity to the participants. We also only used Android Wear participants and limited each group to 10 members, which may not be adequate. We were also aware that the age range of our participants was relatively narrow. Most of our participants in this study were young adults; hence, our results may not apply to older adults. Furthermore, comparing active calories burned as an absolute value could have negatively influenced the reliability of the results because of potential confounding variables such as gender, weight, and height.

The language of our questions could be improved by being more neutral and consistent. For example, we occasionally used *task* to refer to the app instead of the more common term *game*, or for EMIC, we used *items* and *activities* for the same purpose. Although these terms could have caused some confusion, which we will improve in the future, we did not receive any negative feedback and do not believe that the inconsistencies significantly affected our findings.

### Conclusions

In this paper, we proposed a system for personalized fitness assistants using gamification and continuous player modeling and reported on a long-term study that investigates the effectiveness of our proposed system. Our findings show that it is possible to provide personalized activity recommendations by continuously updating a player model based on activity tracking. Our study also shows the positive effect of this modeling and gamification on user engagement and overall activity. These findings can be used to inform the design of personalized and gamified recommender systems in health and fitness and potentially other apps, as they highlight the role of an adaptive model and gamification as long-term and short-term factors, respectively. This research opens opportunities for future work, especially in the area of exploring more gameplay features, adding a personalized storyline, multiplayer gamification, better activity recognition, suggestion models, and evaluation with a larger and more diverse sample.

## Appendix

Multimedia Appendix 1 Prestudy questionnaire for collecting general information.

Multimedia Appendix 2 Poststudy questionnaire for collecting general feedback. EMIC: European Microsoft Innovation Center; IMI: Intrinsic Motivation Inventory.

Multimedia Appendix 3 Intrinsic Motivation Inventory for evaluating the level of enjoyment related to the game experience.

Multimedia Appendix 4 European Microsoft Innovation Center recommender system evaluation measurement tool.

Multimedia Appendix 5 Eight Colors of Fitness activity suggestions.

## Abbreviations

ANOVA: analysis of variance
API: application programming interface
EMIC: European Microsoft Innovation Center
GRPAH: Global Recommendation on Physical Activity for Health
HSD: honestly significant difference
IMI: Intrinsic Motivation Inventory
MBTI: Myers-Briggs–Type Indicator
MC: mean score for control group
MF: mean score for full group
MG: mean score for gamified group
MP: mean score for personalized group
SDC: SD for control group
SDF: SD for full group
SDG: SD for gamified group
SDP: SD for personalized group
UI: user interface
VR: virtual reality
