# Supplementary material for: Effects of a Personalized Fitness Recommender System Using Gamification and Continuous Player Modeling: System Design and Long-Term Validation Study
Source: JMIR Serious Games. 2020 Nov 17;8(4):e19968. doi: 10.2196/19968 (PMC7708084; doi:10.2196/19968)

A.1 Questionnaire 1 (pre-study):

Part I:

Please indicate the following:

Age: _______________

Gender: _______________

Weight: _______________

Height: _______________

Average hours of exercise per week: _______________

Average hours spent per week playing computer/mobile games: _____________

Activity trackers or wearables owned: ___________________________________________

Part II (Hexad gamification user types):

| 1 | 2 | 3 | 4 | 5 | 6 | 7 |
| --- | --- | --- | --- | --- | --- | --- |
| Strongly disagree | Disagree | Somewhat disagree | Neither agree or disagree | Somewhat agree | Agree | Strongly agree |

For each of the following statements, please indicate your level of agreement. Please circle your response.

| 1 | Interacting with others is important to me. | 1 2 3 4 5 6 7 |
| --- | --- | --- |
| 2 | It makes me happy if I am able to help others. | 1 2 3 4 5 6 7 |
| 3 | It is important to me to follow my own path. | 1 2 3 4 5 6 7 |
| 4 | I like being part of a team. | 1 2 3 4 5 6 7 |
| 5 | I like to provoke. | 1 2 3 4 5 6 7 |
| 6 | I am very ambitious. | 1 2 3 4 5 6 7 |
| 7 | I like competitions where a prize can be won. | 1 2 3 4 5 6 7 |
| 8 | It is important to me to feel like I am part of a community. | 1 2 3 4 5 6 7 |
| 9 | I often let my curiosity guide me. | 1 2 3 4 5 6 7 |
| 10 | I feel good taking on the role of a mentor. | 1 2 3 4 5 6 7 |
| 11 | I like to question the status quo. | 1 2 3 4 5 6 7 |
| 12 | It is more fun to be with others than by myself. | 1 2 3 4 5 6 7 |
| 13 | Rewards are a great way to motivate me. | 1 2 3 4 5 6 7 |
| 14 | I like to try new things. | 1 2 3 4 5 6 7 |
| 15 | I like defeating obstacles. | 1 2 3 4 5 6 7 |
| 16 | I look out for my own interests. | 1 2 3 4 5 6 7 |
| 17 | I like helping others to orient themselves in new situations. | 1 2 3 4 5 6 7 |
| 18 | I see myself as a rebel. | 1 2 3 4 5 6 7 |
| 19 | I enjoy group activities. | 1 2 3 4 5 6 7 |
| 20 | It is important to me to always carry out my tasks completely. | 1 2 3 4 5 6 7 |
| 21 | I prefer setting my own goals. | 1 2 3 4 5 6 7 |
| 22 | I dislike following rules. | 1 2 3 4 5 6 7 |
| 23 | I like sharing my knowledge. | 1 2 3 4 5 6 7 |
| 24 | It is difficult for me to let go of a problem before I have found a solution. | 1 2 3 4 5 6 7 |
| 25 | Return of investment is important to me. | 1 2 3 4 5 6 7 |
| 26 | Being independent is important to me. | 1 2 3 4 5 6 7 |
| 27 | I like mastering difficult tasks. | 1 2 3 4 5 6 7 |
| 28 | The well-being of others is important to me. | 1 2 3 4 5 6 7 |
| 29 | I like to take changing things into my own hands. | 1 2 3 4 5 6 7 |
| 30 | If the reward is sufficient I will put in the effort. | 1 2 3 4 5 6 7 |

Part III (8-colors types):


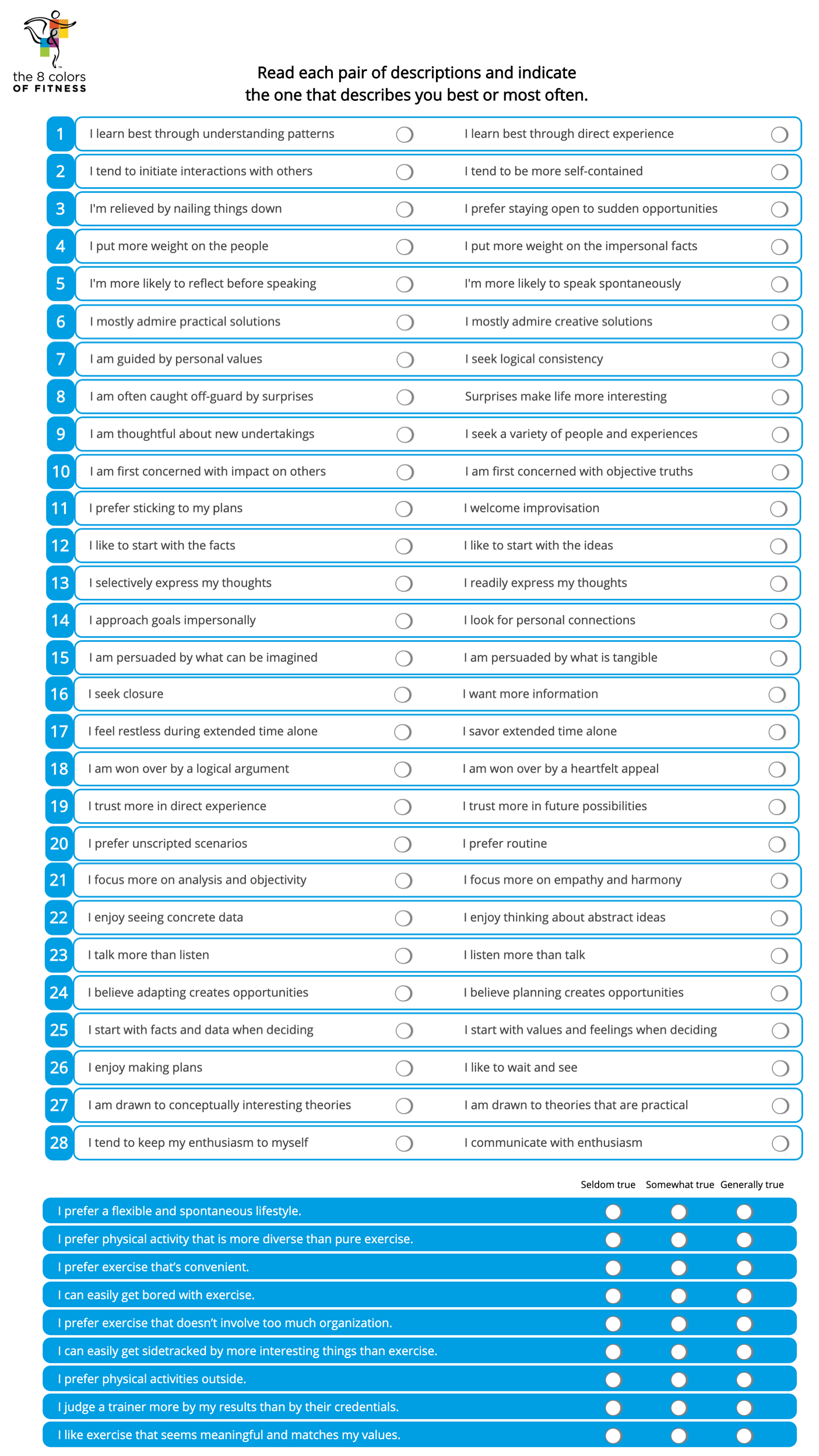

Supplement: Multimedia Appendix 1 [file games_v8i4e19968_app1.docx]
