# Supplementary material for: Effects of a Personalized Fitness Recommender System Using Gamification and Continuous Player Modeling: System Design and Long-Term Validation Study
Source: JMIR Serious Games. 2020 Nov 17;8(4):e19968. doi: 10.2196/19968 (PMC7708084; doi:10.2196/19968)
Supplement: Multimedia Appendix 2 [file games_v8i4e19968_app2.docx]

A.2 Questionnaire 2 (post-study)

For each of the following statements, please indicate your level of agreement. Please circle your response.

| 1 | 2 | 3 | 4 | 5 | 6 | 7 |
| --- | --- | --- | --- | --- | --- | --- |
| Strongly disagree | Disagree | Somewhat disagree | Neither agree or disagree | Somewhat agree | Agree | Strongly agree |

Part I (The 8 color of fitness activity suggestions)

1. Do you find this kind of application motivating for exercise? (overall motivation)

1 2 3 4 5 6 7

2. How would you rate your overall satisfaction with this application? (overall satisfaction)

1 2 3 4 5 6 7

3. In general, what is your level of preference for using this type of application for exercise over regular exercise?

1 2 3 4 5 6 7 (overall preference)

Part II (IMI questionnaire) (see A.3)

Part III (EMIC questionnaire) (see A.4)

Please provide your suggestions on how you think this system could be improved: ______________________________________________________________________________
