# Supplementary material for: Effects of a Personalized Fitness Recommender System Using Gamification and Continuous Player Modeling: System Design and Long-Term Validation Study
Source: JMIR Serious Games. 2020 Nov 17;8(4):e19968. doi: 10.2196/19968 (PMC7708084; doi:10.2196/19968)
Supplement: Multimedia Appendix 3 [file games_v8i4e19968_app3.docx]

A.3 Intrinsic Motivation Inventory (Using the same Likert scales)

Interest/Enjoyment was measured by seven sentences:

- I enjoyed playing this game very much;
- This game was fun to play;
- I thought this was a boring game (reverse coded);
- This game did not hold my attention at all (reverse coded);
- I would describe this game as very interesting;
- I thought this game was quite enjoyable;
- While I was playing this game, I was thinking about how much I enjoyed it.

Perceived Competence was measured by six sentences:

- I think I am pretty good at this game;
- I think I did pretty well at this game, compared to other players;
- After playing this game for a while, I felt pretty competent;
- I am satisfied with my performance at this time;
- I was pretty skilled at this game;
- This was a game that I couldn’t do very well (reverse coded).

Effort/Importance was measured by five sentences:

- I put a lot of effort into this game;
- I didn’t try very hard to do well at this game (reverse coded);
- I tried very hard at this game;
- It was important to me to do well at this game.
- I didn’t put much energy into this game (reverse coded).

Pressure/Tension was measured by five sentences:

- I did not feel nervous at all while doing this (reverse coded);
- I felt very tense while doing this activity;
- I was very relaxed in doing these (reverse coded);
- I was anxious while working on this task;
- I felt pressured while doing these.

Value/Usefulness was measured by 7 statements:

- I believe this game could be of some value to me;
- I think that playing this game is useful for keeping me active;
- I think this is important to do because it can motivate me to engage in exercise;
- I would be willing to lay it again because it has some value to me;
- I think playing this game could help me to exercise more often;
- I think playing this game could be beneficial to me;
- I think this is an important game.
