# Supplementary material for: Effects of a Personalized Fitness Recommender System Using Gamification and Continuous Player Modeling: System Design and Long-Term Validation Study
Source: JMIR Serious Games. 2020 Nov 17;8(4):e19968. doi: 10.2196/19968 (PMC7708084; doi:10.2196/19968)
Supplement: Multimedia Appendix 4 [file games_v8i4e19968_app4.docx]

A.4 EMIC (European Microsoft Innovation Center) recommender system evaluation measurement (Using the same Likert scales)

Perceived recommendation quality was measured by seven sentences:

- I like the activities recommended by the system;
- The recommended activities were well-chosen;
- The recommended activities fitted my preference;
- The recommended activities were relevant;
- The system recommended too many bad activities (reverse coded);
- I didn’t like any of the recommended activities (reverse coded);
- The activities I selected were “the best among the worst” (reverse coded).

Perceived system effectiveness was measured by six sentences:

- I would recommend the system to others;
- The system is useless (reverse coded);
- The system makes me more aware of my choice options;
- I make better choices with the system;
- I can find better items without the help of the system (reverse coded);
- I can find better items using the recommender system.

General trust in technology was measured by four sentences:

- Technology never works (reverse coded);
- I’m less confident when I use technology (reverse coded);
- The usefulness of technology is highly overrated (reverse coded);
- Technology may cause harm to people (reverse coded).

System-specific privacy concern was measured by four sentences:

- I’m afraid the system disclosed private information about me (reverse coded);
- The system invades my privacy (reverse coded);
- I feel confident that the system respects my privacy;
- I’m uncomfortable providing private data to the system (reverse coded).
