# Supplementary material for: Effects of a Personalized Fitness Recommender System Using Gamification and Continuous Player Modeling: System Design and Long-Term Validation Study
Source: JMIR Serious Games. 2020 Nov 17;8(4):e19968. doi: 10.2196/19968 (PMC7708084; doi:10.2196/19968)
Supplement: Multimedia Appendix 5 [file games_v8i4e19968_app5.docx]

A.5 The 8 color of fitness activity suggestions (8Colors, 2008)

| Blues | Rowing, running, walking, yoga, interval training |
| --- | --- |
| Golds | Swimming, Pilates, yoga, tennis, one-on-one personal training, hiking |
| Reds | Basketball, tennis, racquetball, in-line skating, Frisbee, mountain biking, soccer, skiing |
| Greens | Hiking, orienteering, backpacking, mountain or road cycling, kayaking, rock climbing, windsurfing |
| Silvers | Tai chi, bicycling or jogging with a group, Zumba, Nia, yoga, group cycling |
| Saffrons | Salsa, belly, ballroom, jazz, folk |
| Whites | Hiking, running, yoga, cardio, gym strength training |
| Purples | Lap swimming, cardio and strength training, running, cycling |
